# Exploring Whether Iron Sequestration within the CNS of Patients with Alzheimer’s Disease Causes a Functional Iron Deficiency That Advances Neurodegeneration

**DOI:** 10.3390/brainsci13030511

**Published:** 2023-03-18

**Authors:** Steven M. LeVine, Sheila Tsau, Sumedha Gunewardena

**Affiliations:** Department of Cell Biology and Physiology, University of Kansas Medical Center, Kansas City, KS 66160, USA

**Keywords:** Alzheimer’s disease, anemia, heme, histochemistry, iron, mitochondria, olfactory bulb, PITRM1

## Abstract

The involvement of iron in the pathogenesis of Alzheimer’s disease (AD) may be multifaceted. Besides potentially inducing oxidative damage, the bioavailability of iron may be limited within the central nervous system, creating a functionally iron-deficient state. By comparing staining results from baseline and modified iron histochemical protocols, iron was found to be more tightly bound within cortical sections from patients with high levels of AD pathology compared to subjects with a diagnosis of something other than AD. To begin examining whether the bound iron could cause a functional iron deficiency, a protein-coding gene expression dataset of initial, middle, and advanced stages of AD from olfactory bulb tissue was analyzed for iron-related processes with an emphasis on anemia-related changes in initial AD to capture early pathogenic events. Indeed, anemia-related processes had statistically significant alterations, and the significance of these changes exceeded those for AD-related processes. Other changes in patients with initial AD included the expressions of transcripts with iron-responsive elements and for genes encoding proteins for iron transport and mitochondrial-related processes. In the latter category, there was a decreased expression for the gene encoding pitrilysin metallopeptidase 1 (PITRM1). Other studies have shown that PITRM1 has an altered activity in patients with AD and is associated with pathological changes in this disease. Analysis of a gene expression dataset from PITRM1-deficient or sufficient organoids also revealed statistically significant changes in anemia-like processes. These findings, together with supporting evidence from the literature, raise the possibility that a pathogenic mechanism of AD could be a functional deficiency of iron contributing to neurodegeneration.

## 1. Introduction

A major tenet guiding drug development efforts for Alzheimer’s disease (AD) has been the amyloid hypothesis [1,2,3]. This hypothesis states that the accumulation of amyloid-β causes a cascade of events leading to neuronal toxicity. This in turn would result in the signs and symptoms of AD. This supposition led to the reasoning that disrupting the formation, aggregation, and/or deposition of amyloid-β would be an effective therapeutic approach. Clinical trials directed against amyloid-β produced results that have had mixed results, i.e., from causing adverse effects to providing no beneficial effects despite effectively reducing the amyloid burden, to slowing some cognitive decline [3,4,5,6,7]. Because of the inability to substantially alter the disease course, greater consideration is being given to the view that AD is a multifactorial disease that can involve pathogenic pathways that function independent of and/or together with amyloid-β [3,4,8,9].

Various pathogenic mechanisms have been proposed to work in conjunction with amyloid-β accumulation to help drive disease. These include the development of tau tangles, activated microglia producing proinflammatory mediators, mitochondrial dysfunction, and disruption of quality control mechanisms for proteins (e.g., autophagy, ubiquitin-proteasome) [4,8,10,11,12]. In addition, the ApoE genotype affects multiple components of pathogenesis (e.g., deposition and clearance of amyloid-β, tau hyperphosphorylation, blood–brain barrier dysfunction, and neuroinflammation) [3,13]. Besides these mechanisms, iron has been implicated in the pathogenesis of AD. For example, iron accumulates in the central nervous system (CNS) of patients with AD, and this accumulation is associated with cognitive decline and atrophy in AD [14,15,16,17,18]. Pathogenic mechanisms involving iron include facilitating the aggregation and toxicity of amyloid-β, augmenting oxidative stress, and possibly causing neuronal death via ferroptosis [11,17,19,20,21,22,23]. The presumed role of iron in AD pathogenesis, however, may be missing key elements or based on an erroneous premise.

A conceptual framework for presumed pathogenic pathways is a direct induction of a deleterious event, e.g., iron accumulation leading to oxidative stress and ferroptosis. Here, we put forward an indirect mechanism that may be relevant for developing a more comprehensive understanding of AD pathogenesis. This indirect mechanism involves a functional deficiency as a contributing factor accounting for downstream deleterious effects. For example, although iron accumulates in the CNS of patients with AD, much of this iron is bound to plaques and tangles, particularly in advanced stages of disease [24,25,26,27,28,29], which could render it unavailable for biochemical reactions that are dependent on iron. Since cellular functions involving iron are numerous (2% of genes in humans encode for a protein that utilizes iron) and they perform essential processes (e.g., electron transport chain, catalysis, oxygen transport) [30], the inability to adequately meet the demand for these reactions could compromise neuronal health and ultimately lead to neurodegeneration.

In this paper we present preliminary evidence that serves as the basis for the hypothesis: In the CNS of patients with AD, iron becomes sequestered, e.g., by binding to amyloid-β and tau, which causes a functional deficiency of iron leading to essential biochemical processes becoming impaired, thereby contributing to neuronal death. In addition to iron, the hypothesis can be expanded to include other metals (e.g., copper, magnesium, zinc) which could become sequestered by various protein aggregates, leading to functional deficiencies, e.g., not allowing biochemical processes to keep up with demands, resulting in neurodegeneration in various diseases of the CNS.

## 2. Materials and Methods

### 2.1. Human CNS Tissue

Deidentified 6 µm-thick paraffin sections were prepared from the frontal cortex of pathologically characterized postmortem brains (8 AD, 6 non-AD) by the Neuropathology Core of the University of Kansas Alzheimer’s Disease Research Center. AD cases had high levels of neuropathologic changes (e.g., A3, B3, and C3) [31] characteristic of advanced disease. Non-AD cases were identified as having adenocarcinoma, multiple infarcts, frontotemporal lobar degeneration, meningioma, etc., and in two of these cases, low AD neuropathological changes (i.e., A1, B1, C0 and A2, B1, C1) were also noted.

### 2.2. Iron Histochemical Staining

Slides were processed using conditions as previously described [32] to obtain a baseline stain. Briefly, for the baseline staining condition, slides were deparaffinized using SafeClear (Fisher Scientific, Waltham, MA, USA, catalog number 23314629) 2 × 3 min; briefly tapped to remove excess fluid; and incubated for 60 min in a solution of 50% acetone, 1% potassium ferrocyanide trihydrate (Millipore Sigma, Burlington, MA, USA, catalog number P3289-100G), and 0.05 N HCl (final concentrations) prepared fresh before use as follows: 80 mL acetone mixed with a solution of 40 mL of deionized filtered (df) H_2_O containing 1.6 g potassium ferrocyanide, followed by mixing the combination with an acid solution of 32 mL dfH_2_O with 8 mL 1N HCl (note, the addition and gentle stirring of the acid solution should clear the cloudy white appearance of the incubation solution). Control slides were incubated in the same solution but without the potassium ferrocyanide. During the 60 min incubation, the staining solution was carefully mixed numerous times to offset any settling. Following staining, slides were washed with multiple changes of tap water and then incubated for 30 min in a solution containing 40 mg 3,3′-diaminobenzidine tetrahydrochloride (Millipore Sigma, catalog number D5905) in 160 mL Tris-HCl pH 7.6 (Millipore Sigma, catalog number 94158-10TAB) with 640 µl 30% hydrogen peroxide. Slides were then washed with multiple changes of tap water, followed by graded EtOH, SafeClear, and a permanent mounting media (Millipore Sigma, catalog number 03989).

For each case studied, additional slides of nearby sections were stained with harsher staining conditions. These slides were processed as described above for the baseline staining condition except that the 40 mL acid solution was composed of 28 mL dfH_2_O with 4 mL EtOH and 8 mL 1N HCl. In addition, at 30 min after the start of incubation, an additional 2 mL of 1N HCl was carefully mixed into the staining solution. This was repeated at 60 min, i.e., an additional 2 mL of 1N HCl was carefully mixed into the staining solution, and the total incubation time in the staining solution was increased to 90 min.

### 2.3. Image Analysis of Frontal Cortex Areas

Images were acquired using a Nikon (Tokyo, Japan) Eclipse 80i microscope equipped with a Nikon Digital Sight DS-Fi3 camera, Nikon NIS-D software, and a 10× Plan Fluor (NA 0.30) objective. Microscope settings included the use of neutral density filters together with a maximum brightness setting and condenser focus followed by fully opening the diaphragm. For each patient’s baseline stained slide, a white balance with the gain set at 1.0 was obtained, and the exposure time was adjusted so that the LUT bandwidths were centered at 200 for the separated red, green, and blue colors. After collection of images from the baseline stained slide (described below), the exposure setting was used for collection of images from the matching harshly stained slide. This process was repeated for each case.

Starting with the baseline stained slide, 3 images were collected, using a 10× objective, from the center of the cortical width and saved as .tif files. Then, 3 images were collected from matching locations on the harshly stained slide. The selection of cortical locations for image collection was performed in a blinded manner and based on areas free of artifacts and with representative staining. These locations were initially identified on scanned images collected using a Plan Apo Lambda 4× objective (NA 0.2) on a Nikon Eclipse High Content Analysis system comprising a Ti-E inverted motorized microscope with perfect focus and a Prior plate loader. Automated imaging was set up with Jobs in Nikon NIS-Elements Advanced Research software. For each slide, final scanned images were tiled from 4× images.

Images collected using the 10× Plan Fluor (NA 0.30) objective were analyzed with ImageJ 2.1.0/v1.53c software (with Fiji) (NIH, Bethesda, MD, USA). The images were converted to an 8-bit (grayscale) file. After examination of different thresholds, a setting of 95 (range 0–255, with 0 as the darkest setting and 255 as the lightest setting) was identified that optimally captured cellular (e.g., neuronal) staining. In addition, for each baseline slide, a threshold was identified that best approximated the staining area to be 5% (4.74–5.21%) of the field. Then, this threshold setting was used for processing images from both baseline stained slides and matching images from harshly stained slides. Thresholding converted the image to a binary file which was processed with a watershed function followed by an analyze particles operation using the size set at 0.00—infinity pixels and circularity set at 0.00–1.00 to determine the percent area of staining. For cell counts, after the watershed process, the analyze particles operation had the size set at 250—infinity pixels and circularity set at 0.00–1.00.

For the 3 locations for each case, the number of instances the harshly stained slide had a greater percent stained area than that for the matching area on the baseline stained slide was determined. Thus, each case received a value of 0, 1, 2, or 3, with 3 representing all three locations having greater percent areas on the slide stained with harsh conditions. Data were analyzed using the two-tailed Mann–Whitney test (GraphPad Prism, version 6.07) with *p* ≤ 0.05 as statistically significant.

### 2.4. Bioinformatics

Transcriptomic data from the human olfactory bulb during AD evolution were obtained by reanalyzing the published [33] microarray GEO dataset (GSE93885). The microarrays were background corrected, normalized, and gene-level summarized using the Robust Multichip Average (RMA) procedure [34]. The resulting log (base 2) transformed signal intensities were used in the R limma package [35] to obtain differential gene expression statistics. The raw *p* values were adjusted for multiple hypothesis testing by the Benjamini and Hochberg [36] procedure, giving a false discovery rate (FDR). Furthermore, the published [37] single cell RNA-sequencing GEO dataset (GSE147047) was interrogated to understand the effect of PITRM1 on AD. We examined the single cell data for iron deficiency markers, both at a cluster level on the 25 clusters identified by the authors and as a whole.

Genes associated with AD and iron dyshomeostasis were identified from several databases including iron-responsive elements (IRE) [38], the human iron proteome [30], the UniProt database [39] searched under the accession “KW-0408”, and the Ingenuity Pathway Analysis (IPA) software’s (Qiagen) bio-profiler. The significance of a cluster of differentially expressed genes belonging to a certain functional category was measured using the hypergeometric test which measures the significance of the overlap between the perturbed genes and genes belonging to the functional category. Small *p* values imply a selective preference for the functional category among the perturbed genes (i.e., less likely to be a chance occurrence). The hypergeometric *p* value was calculated separately for the upregulated genes, downregulated genes, and perturbed genes regardless of the direction of expression. Genes with an absolute fold change ≥ 1.5 and a *p* value ≤ 0.05 were considered significant in a perturbed cluster of genes belonging to a functional category.

## 3. Results and Discussion

### 3.1. Iron Histochemistry

**Figure 1:**
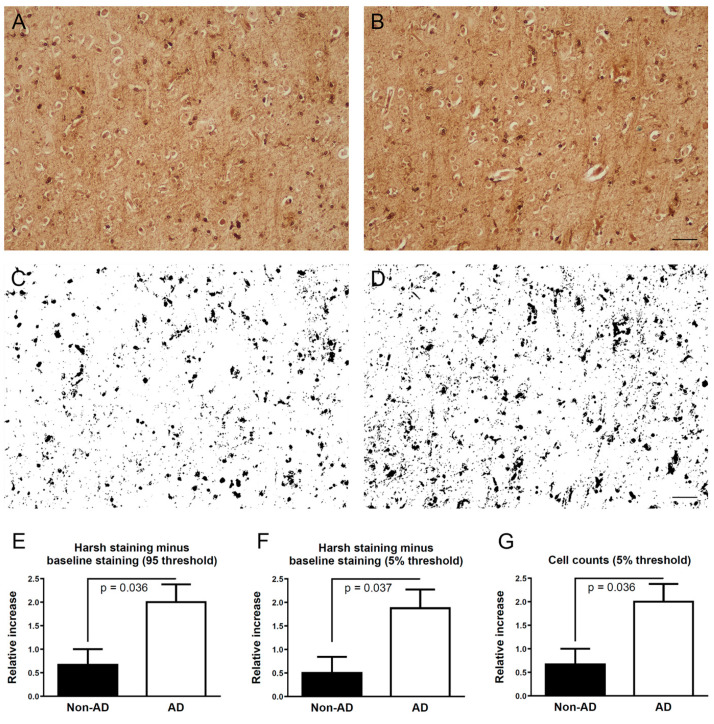
Comparison of staining using baseline and harsh conditions for iron histochemistry. (**A**) A section of frontal cortex from a patient with AD stained with baseline conditions reveals neuropil and cellular staining; (**B**) The matching cortical region of the same patient as in (**A**) but stained using harsh conditions reveals darker overall staining than in panel (**A**); (**C**) Thresholding of baseline staining from the image in (**A**) reveals cellular and neuropil staining that crossed the threshold setting of 95 (range 0–255); (**D**) Thresholding of staining with harsh conditions from the image in (**B**) reveals cellular and neuropil staining that crossed the threshold setting of 95. Note the greater abundance of staining in (**D**) than in (**C**); (**E**,**F**) The relative abundance of stained area (harsh staining minus baseline staining) using a threshold of 95 (**E**) or 5% (**F**) from 3 cortical regions per patient; (**G**) The relative abundance of stained cells (harsh staining minus baseline staining) using a threshold of 5% from 3 cortical regions per patient. Two-tailed Mann–Whitney U test, mean with standard error of the mean. Non-AD subjects (n = 6), patients with AD (n = 8). Bar in B and D = 50 µm.

In the frontal cortex of AD and non-AD cases, both baseline and harsh staining conditions revealed cellular and neuropil staining, which ranged from light, i.e., matching background levels, to dark brown (Figure 1A,B). Comparison of matched cortical areas between the baseline slides and harshly stained slides revealed that patients with AD had a greater relative area and greater cellular numbers stained with harsh conditions compared to non-AD patients (Figure 1C–G). Thus, the harsh staining conditions made more iron available for staining in the AD cases than in non-AD cases.

### 3.2. Selection of a Suitable AD Dataset

To begin exploring the idea that the tightly bound iron present in AD cases causes a shortage of iron, i.e., limiting the availability of iron for cellular functions, a gene expression dataset from patients with and without AD was reasoned to contain pertinent information. Several criteria were considered for the selection of the dataset for analysis. (1) Did the dataset include analysis of tissue at different stages of AD, particularly the early stage? The reasoning for emphasizing the early stage was that it is more likely to capture ongoing pathogenic mechanisms, while the late stages of AD are more likely to reflect the consequences of neurodegeneration, e.g., astrocyte gliosis; (2) Was the dataset generated from a relevant area of the CNS that is likely affected early in the disease course? Deficits in olfaction can be an early symptom of AD [40], and like the hippocampus, which is a key pathological site in AD, the olfactory bulb is involved early and throughout the disease process, e.g., displaying both initial Braak’s stages as well as atrophy [41,42]. Furthermore, like the hippocampus, the olfactory bulb continues to undergo neurogenesis into adulthood [43]. Thus, a study of the olfactory bulb has the potential to reveal relevant information about the pathogenesis of AD that could be missed in the analyses of other regions of the CNS; (3) Does the dataset reflect the differentially expressed protein-coding transcripts between control and diseased tissue? The dataset generated in the study by Lachen-Montes et al. [33], GSE93885, met all three of these criteria, e.g., it generated data on protein-coding transcripts from a relevant structure for AD at different stages of disease; thus, in the present study it was analyzed for pathways that involve the utilization of iron.

In order to best capture ongoing pathogenic events in the relevant CNS region, the emphasis for the analysis was on the initial stage of AD vs. control cases. To confirm that this comparison captured changes pertinent to AD, genes with ≥ or ≤ 1.5 fold difference in expression, with a *p* ≤ 0.05, were identified using the IPA BioProfiler category of “Alzheimer’s disease”. Indeed, the hypergeometric *p* values were statistically significant for both upregulated and downregulated genes in the category of AD (Table 1).

### 3.3. Anemia-Like Responses in AD Olfactory Bulb Dataset

**Figure 2:**
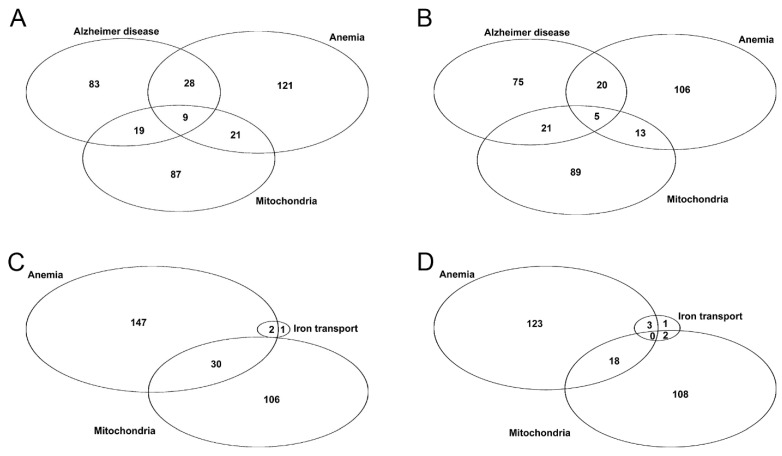
For patients with initial AD vs. control subjects, Venn diagrams illustrating the number of genes with overlapping expression differences in the categories of AD, anemia, and mitochondria for (**A**) upregulated; and (**B**) downregulated genes. Venn diagrams illustrating the number of genes with overlapping expression differences in the categories of iron transport, anemia, and mitochondria for (**C**) upregulated; and (**D**) downregulated genes.

Since histochemical findings revealed that iron was being sequestered, the principal question was whether there are alterations in the expression of genes encoding proteins that have a role in anemia-related processes during AD. The reasoning was that if iron was unavailable in neurons in AD, then the response in the CNS could resemble that occurring in tissues outside the CNS during an anemic state, which is often caused by iron deficiency. Thus, to explore if anemia-related genes are affected in early AD, the genes in the IPA BioProfiler category of “Anemia” were analyzed. Both upregulated and downregulated genes in the anemia category had statistically significant hypergeometric *p* values for initial AD vs. non-AD (Table 1) with 26.6 and 20.7% of the up and downregulated AD genes (Table 1, Supplemental Table S1), respectively, overlapping with those in the anemia category (Figure 2A,B). It is noteworthy that, as indicated by the hypergeometric *p* values, the associations for the category of anemia were even more significant than those within the category of AD (Table 1). This finding indicates that the early pathogenic changes that occur in AD have the appearance of anemia-like responses, at least in the olfactory bulb.

### 3.4. Altered Expressions Related to Cellular Iron Transport in Early AD

The expression of genes encoding proteins involved with the transport of iron could be altered during conditions perceived as being limited for iron availability. In the olfactory bulb of patients with initial AD vs. control subjects, 66.7% and 50% of the up and downregulated iron transport genes (Table 1, Supplemental Table S1), respectively, overlapped with those in the anemia category (Figure 2C,D). The expression of transcripts for the gene for transferrin (TF) are upregulated in patients with initial AD vs. control subjects, and it is not until patients have an advanced stage of disease that the transferrin receptor 2 (TFR2) becomes significantly downregulated (Supplemental Tables S1 and S2). The expression of the gene SLC11A2, which encodes for the iron uptake transporter divalent metal transporter 1, was upregulated in patients with initial AD vs. control subjects, but the change did not reach significance (*p* = 0.084) (Supplemental Table S2). The expression of a transcript for the SLC40A1 gene, encoding for the iron export protein ferroportin, is downregulated in the olfactory bulb of patients with initial AD vs. control subjects. This reduction was not detected (not significant) for other stages of AD (Supplemental Tables S1 and S2), or in other studies of AD CNS tissue that did not examine early AD [44,45], which highlights the importance of performing studies on early stages of AD. In fact, changes in the expression of genes related to the management of iron occurred predominantly in patients with initial AD, and substantially less so in patients with middle or advanced AD (Supplemental Table S2). In addition, the expression of transcripts for the gene for ceruloplasmin (CP), a ferroxidase that facilitates iron uptake and efflux, is significantly upregulated in initial, middle, and advanced AD (Supplemental Tables S1 and S2), and together with transferrin (discussed above), it may favor processes leading to iron uptake [46]. Together, these changes can be viewed as being consistent with cells sensing a limited availability of iron. In contrast, the expression of the gene for STEAP3, a ferrireductase which facilitates iron uptake, and the gene for ferritin heavy chain 1 (FTH1), a ferroxidase involved in iron storage, have transcripts that are upregulated and/or downregulated in patients with initial AD vs. control subjects (Supplemental Tables S1 and S2), which could suggest that a more complicated management of iron is taking place.

Although other studies found that the expression of the SLC40A1 gene was not altered in the cortex of patients with AD, or in the hippocampus of APP/PS1 mice, the accompanying protein levels of ferroportin were reduced in AD subjects as well as in APP-tg (Tg2576) and APP/PS1 mice [44,45], suggesting increased internalization and degradation of the protein [23]. Amyloid precursor protein (APP) is thought to help stabilize ferroportin, facilitating iron efflux, although other possible mechanisms have been put forward [38]. Overexpression of APP in cultured human neuroblastoma cells (SH-SY5Y) led to decreased iron levels, reduced activity of the antioxidant enzyme catalase, which utilizes four hemes, and increased level of reactive oxygen species [47]. However, amyloidogenic processing of APP may promote internalization and degradation of ferroportin [23,48]. Thus, a decreased expression and function of ferroportin can lead to iron accumulation, which may help establish oxidative conditions for ferroptosis, which has been thought to contribute to the development of behavioral and pathological changes associated with AD progression [23,45]. However, in patients with initial AD, the expressions of transcripts for the gene for ATF6, a transcription factor induced by the unfolded protein response, were downregulated (Supplemental Tables S1 and S2). Since inhibition of ATF6 potentially blocks ferroptosis [49], the downregulation of ATF6 gene expression may help offset this mechanism of cell death. In addition, IRP2 facilitates both iron acquisition by neurons [50] and ferroptosis [51,52]; and since the expressions of transcripts for the gene for IRP2, i.e., IREB2, are downregulated in patients with initial AD (Supplemental Tables S1 and S2), these responses may be limited during the early stage of disease. Interestingly, olfactory bulb transcripts that bind IRP2 or IRP1, i.e., with a 3′ iron response element (IRE) or a 5′ IRE [38], had pronounced statistical differences in patients with early AD (Table 1). Additional genes that encode iron transport proteins within mitochondria are described in the next section.

### 3.5. Altered Expressions Related to Mitochondrial Iron Transport in Early AD

For patients with initial AD vs. control subjects, the difference in expression levels of the genes in the category of mitochondria were statistically significant (Table 1), with 22.06% and 14.06% of the up and downregulated mitochondrial genes (Table 1, Supplemental Table S1), respectively, overlapping with those in the anemia category (Figure 2A,B). Iron is used extensively for mitochondrial metabolism and function; therefore, transport and homeostasis of iron within mitochondria is highly regulated [53,54]. The expression of a transcript for mitoferrin 2 (SLC25A28) is upregulated across all stages of AD, while the expression of a transcript for ATP binding cassette subfamily B member 7 (ABCB7) and member 8 (ABCB8) are decreased in the olfactory bulb of initial AD vs. control subjects (Supplemental Tables S1 and S2). This expression profile is consistent with the goal of delivering more iron to the mitochondria, since mitoferrin 2 mediates the uptake of iron by mitochondria, while ABCB7 and ABCB8 are involved with mitochondrial iron export (i.e., the maturation and export of glutathione-coordinated iron–sulfur clusters from the mitochondria and the export of iron from the mitochondria, respectively) [54,55,56,57]. In addition, the expression of transcripts for the gene frataxin (FXN) are upregulated in initial AD vs. control subjects (Supplemental Tables S1 and S2). Since frataxin acts as a chaperon for iron and facilitates the synthesis of heme and formation of iron–sulfur clusters [58], the upregulation of the FXN gene is consistent with a low availability of mitochondrial iron. If iron is sequestered by protein aggregates within mitochondria, e.g., due to deficient PITRM1 discussed below, then the unavailable iron could be perceived as a deficiency of iron leading to impaired mitochondrial function, e.g., deficient cytochrome c oxidase activity (also discussed below).

### 3.6. Changes Related to Mitochondria

Despite the accumulation of iron, we suggested that the deposited iron would not be available for enzymatic use, leading to a functional iron deficiency. This functional deficiency, however, would not be mutually exclusive of oxidative conditions involving iron that leads to ferroptosis. We suggest that a functional iron deficiency is involved with early pathogenic events which can occur in parallel with the accumulation of iron and elevated levels of oxidative stress. As mitochondria become damaged, the effects of a functional iron deficiency on mitochondrial activity could become more acute. Interestingly, the gene expression levels of GSR (i.e., glutathione reductase, which reduces oxidized glutathione disulfide to the sulfhydryl version) and GSTP1 (glutathione S-transferase pi 1, which is involved with detoxification) are elevated in patients with initial AD (Supplemental Tables S1 and S2), and their respective proteins both help protect mitochondria from oxidative stress [59,60].

One of the genes in the mitochondria category that had a transcript with a ≤ 1.5 fold decreased expression in patients with initial AD was PITRM1 (Supplemental Tables S1 and S2), which encodes for pitrilysin metallopeptidase 1 (known as presequence peptidase). This finding is aligned with previous studies that found that both the transcripts and activity of PITRM1 are reduced in AD patients [61,62], although the transcripts were observed in isolated astrocytes and the activity was measured from the mitochondrial matrix collected from the temporal lobe. This mitochondrial Zn-metalloprotease was of particular interest since it processes precursor proteins and digests peptides including amyloid-β [63,64]. Additionally, mice deficient in PITRM1 accumulate APP and amyloid-β [65], and cerebral organoids derived from human-induced pluripotent stem cells that are deficient in PITRM1 have tau pathology, protein aggregates, and neuronal death [37].

### 3.7. Analysis of Dataset from Human Organoids with or without PITRM1

Pérez et al. [37] performed single cell RNA sequencing on PITRM1 deficient and matching control human organoids induced from pluripotent stem cells. An FDR of ≤ 0.05 was used for prefiltering data, resulting in the analyzed data for GSE147047 [37]. We analyzed this dataset for differences in the expression of genes related to pathways involved in iron utilization, again with the primary focus on anemia-related genes. Unlike the study by Lachen-Montes et al. [33], the study by Pérez et al. [37] examined single cells, which can bypass problems associated with the interpretation of data relative to which cell types were expressing the mRNAs as well as the changing proportions of cell types that occur during the course of disease.

When combining all clusters, both upregulated and downregulated genes within the anemia category had statistically significant hypergeometric *p* values (Table 2; last section of Supplemental Table S3). This result suggests that protein aggregation within mitochondria, due to a deficiency in PITRM1, can result in an anemia-like response. The cell types represented by the clusters with statistically significant hypergeometric *p* values for upregulated anemia-related genes were five clusters representing neurons, three clusters representing progenitor cells, and one cluster representing glia (Table 3). The clusters with statistically significant hypergeometric *p* values for downregulated anemia-related genes were one cluster representing neurons and one cluster representing astrocytes (Table 3). Thus, the anemia-like response was predominantly occurring within neurons. Since neurons in PITRM1-deficient organoids undergo degeneration, e.g., become positive for cleaved caspase-3 [37], this raises the possibility that anemia-like events are contributing to the neurodegeneration, and similar events could contribute to the degeneration of neurons that occurs in AD. Interestingly, since the olfactory bulb, like the dentate gyrus, undergoes neurogenesis during adult life [43], the anemia-like response within the progenitor cells raises the possibility that anemia contributes to the impairment of neurogenesis that has been observed during AD [66]. Thus, anemia-like responses might facilitate neurodegeneration and impair neurogenesis, both of which can contribute to atrophy that occurs in the olfactory bulb and hippocampus of patients with AD.

The other category with the most statistically significant hypergeometric *p* value was for 5′ IRE (Table 2). 5′ IRE was upregulated in one neuronal cluster and downregulated in two neuronal clusters, one progenitor cluster, and one glia cluster (Table 3).

### 3.8. Comparison of Datasets

The two datasets, i.e., AD olfactory bulb and PITRM1 organoids, were compared to determine if the anemia-like responses that occur in the PITRM1-deficient organoids are reflective of one or more stages of AD. When comparing the data from the different stages of AD, the initial stage had the best alignment of anemia-related genes compared with PITRM1-deficient organoids, with a statistically significant hypergeometric *p* value for downregulated genes (Table 4).

The middle stage of AD also had a significant hypergeometric *p* value, but this was only if the expression of the genes was allowed to be up or downregulated (Table 4). There was no statistical significance between the expression of genes for the advanced stage of AD with that for PITRM1-deficient organoids (Table 4). Thus, the PITRM1 deficient organoids best correlated with the changes occurring during the initial stages of AD, supporting the concept that these changes are involved early in disease progression.

### 3.9. Hypothesis

In cortical sections from AD patients vs. non-AD patients, we show that by increasing the acid concentration during the histochemical reaction, some iron becomes preferentially stained, indicating that this iron has been tightly bound. In addition, we show that the initial stage of AD leads to changes in the expression of protein-coding transcripts that reflect anemia-like responses, and these responses were also observed in an organoid model with direct relevance to AD, i.e., protein aggregates, tau pathology, and neuronal degeneration. Based on these preliminary findings and associated results from the scientific literature, we put forward the following hypothesis: iron becomes sequestered in the CNS of patients with AD, leading to a functional iron deficiency that results in neuronal degeneration.

### 3.10. Mechanism Leading to a Functional Deficiency

Among the profile of altered enzymes in AD, a decreased activity of PITRM1 could contribute to the formation of amyloid and tau deposits in AD [37,61,63,64,65]. Since both amyloid-β and tau bind iron [24,26,67,68,69,70], they are likely participants in the sequestration of iron in patients with AD. Amyloid-β and tau bind iron through histidine and possibly other residues [28,29,69,71,72]. Another indicator that iron is tightly bound in the presence of amyloid-β is that when CNS tissue was pretreated with acid (prior to staining), or pretreated with a protease detergent solution, iron-positive plaques were still detected in APP/PS1 mice [73], i.e., the pretreatment regimen was not sufficient to remove iron from this site. Amyloid-β around vessels may also be a potential location of iron sequestration since amyloid angiopathy can result in iron deposition [32,74,75], and in the present study, cortical cells had tightly bound iron in patients with AD (Figure 1). Besides binding iron, amyloid-β has been shown to bind heme, which may lead to a functional deficiency of heme, resulting in impairment of mitochondrial activity [76,77,78]. Additionally, findings by Hin et al. [38] raised the prospect that a deficiency of functional ferrous iron in the presence of excess ferric iron contributes to AD pathogenesis, and Belaidi and Bush [79] raised the possibility that iron deficiency could be a mechanism that facilitates neurodegeneration. Deficiencies of both heme and iron have been linked with induction of ATF4 and eIF2α phosphorylation [80], which can limit both protein translation and memory formation [81].

Copper and zinc also bind amyloid-β and tau [25,68,82,83]. Thus, besides iron, it is possible that other metals could become sequestered, leading to a functional deficiency for related biochemical processes, thereby contributing to neurodegeneration. Interestingly, a functional deficiency for zinc and a dietary deficiency in copper were previously hypothesized as pathogenic mechanisms for AD [84,85].

### 3.11. An Example of a Functional Deficiency

Numerous enzymes are potential targets for iron and/or copper deficiency. In humans, there are 398 or more genes that encode for proteins that utilize iron with functions including respiration, lipid synthesis, DNA synthesis, and neurotransmitter synthesis [30,86,87]. Similarly, copper is associated with numerous essential functions including neurotransmitter synthesis, synaptic function, respiration, and cellular defenses [88,89]. In other model systems, e.g., plants and heart, iron deficiency is directly associated with altered mitochondrial function, particularly of electron transport complexes [90,91].

A possible example of a functional deficiency in AD is decreased activity of cytochrome c oxidase (known as mitochondrial complex IV). Multiple studies have established that the activity of this complex, but not necessarily other mitochondrial complexes, is reduced in the CNS of patients with AD [92,93,94,95,96,97]. Impaired cytochrome c oxidase can decrease mitochondrial function [98], which can lead to neurodegeneration [99].

In mammals, cytochrome c oxidase has 14 subunits and has been proposed to utilize 2 iron/heme, 3 copper, 1 magnesium, and 1 zinc for assembly, structure or function [100,101,102]; thus, the unavailability of different metals could impact the activity of this enzyme [103]. For instance, deficiencies in either iron or copper have been associated with impaired cytochrome c oxidase activity [91,104,105,106]. In the heart, the reduction in enzymatic activity in response to iron deficiency was not related to changes in the amount of cytochrome c oxidase protein levels, or the number or morphology of mitochondria [91], raising the possibility that a functional deficiency accounted for decreased activity. In AD, altered kinetic activity of cytochrome c oxidase was observed [107,108]. While this change in activity was suggested to involve sites on the complex other than those utilizing cytochrome aa3 [107,108], which involves two heme-a groups, this point was questioned, since it was noted that in the earlier study [107], the reduction in the level of heme-a was 22%, although this reduction was not statistically significant [76]. It was suggested that a deficiency in heme leads to a decrease in heme-a and a reduction in cytochrome c oxidase activity [76]. In a related study, a non-significant 26% reduction in heme-a was observed in the temporal lobe of patients with AD vs. control subjects [109], but a subsequent study found a significant increase in heme-a in the frontal cortex of patients with AD [110]. A possible explanation for the increase in heme-a was an elevated level of mitochondrial damage in patients with AD [110]. A consideration that was not addressed in earlier studies [107,109,110] was the effect of the AD disease stage on heme-a levels or enzyme activity. In addition, different brain regions and different cellular compartments could affect the outcome of these studies [76,110].

If a change in activity is present early, then it may contribute to the disease process rather than occurring as a consequence. From the analysis of the olfactory bulb dataset, the expression of COX15, which encodes for heme A synthase, is upregulated in patients with initial or moderate AD, but did not reach statistical significance in advanced AD, compared to control subjects (Supplemental Tables S1 and S2). An elevation of COX15 mRNA had been observed previously in hippocampal tissue from patients with AD [111]. In contrast to COX15, the expression of a transcript for the gene COX10, which encodes for heme A: farnesyltransferase, is downregulated in patients with initial (or advanced) AD compared to control subjects (Supplemental Tables S1 and S2). In COX10 conditional knockout mice, alterations in rotarod and ambulatory behavior were observed starting at 3 months of age, and neurodegeneration was observed in the hippocampus and cortex starting at 4 months of age [112]. When the conditional COX10 deficiency was combined with APP/PS1 mice, there was a reduction in both amyloid-β42 and plaque numbers, suggesting that the changes to mitochondrial function in AD are a result of amyloid-β rather than the other way around [113]. This interpretation would be consistent with a previously presented view that amyloid-β binds heme, leading to a functional deficiency of heme and altered mitochondrial function [76,77,78,114]. Together, these data indicate that cytochrome c oxidase function is affected in AD, with alterations to the expression of associated genes starting early in the course of AD.

### 3.12. Senescent Cells and Impaired Ferritinophagy

Cellular senescence has been linked with aging, inflammation, and AD [115]. For example, CNS tissue from AD patients displays an increase in double-stranded breaks [116], impaired degradation and clearance of proteins [117], and other features of senescent cells [115,116,117,118,119]. Interestingly, cellular senescence has been found to lead to intracellular accumulation of iron [120]. In addition, the degradation of ferritin, via ferritinophagy, is impaired in senescent cells, resulting in iron becoming sequestered in ferritin and causing a perceived deficiency of iron [120]. Mitochondrial ferritin might be another source of iron sequestration as its mRNA and protein levels are increased in the temporal lobe of patients with AD [121]. Similar to our hypothesis, increased ferritin and decreased ferritinophagy have been proposed to lead to a functional deficiency of iron by sequestering iron, which in turn would impair mitochondrial function and amplify inflammation, thereby promoting aging and neurodegeneration [122].

### 3.13. Limitations

This study represents an initial exploration into the possibility that iron becomes functionally deficient in patients with AD. There are several limitations to the histochemical study including the small number of subjects and examination of only one CNS region. In addition, other factors that would strengthen subsequent studies would be to compare the effects of ApoE genotypes, analyzing tissue specimens processed from different centers, and closely matching AD and non-AD subjects across demographics. In addition, an array of complementary experiments can probe more deeply into the question of whether a functional iron deficiency exists, and if so, how much of an impact it has on the disease course. Relative to data analyses, it would be interesting to see if similar results are obtained from other datasets, but it is difficult to find datasets that meet the specific requirements used for our study. Furthermore, it would be informative to have data on both the levels and activity of proteins matching the relevant genes with altered expression levels. Thus, the findings presented in this study should be considered preliminary, i.e., they raise the possibility that a functional deficiency is a relevant pathogenic mechanism in AD.

## 4. Conclusions

The present study offers preliminary findings indicating that iron is more tightly bound in cortical tissue from AD patients vs. non-AD subjects, and that early disease activity exhibits gene expression changes that are consistent with responses that are present during an anemia-like state. These findings, together with results presented in the literature, provide the foundation for the hypothesis that during AD, iron becomes sequestered, e.g., due to protein aggregates, which leads to a functional deficiency and ensuing neurodegeneration. If correct, then this would generate the possibility of new therapeutic targets. For instance, limiting the sequestration of iron would improve its availability for enzymatic activities, many of which are essential to neuronal function and survival. Providing additional iron would be unlikely, by itself, to give rise to a benefit, as it too would likely become sequestered, and the extra iron could amplify oxidative damage. Iron chelation has been explored as a therapy for AD. While chelation may help to offset oxidative damage, it would do little to restore a functional iron deficiency and possibly even exacerbate the situation by further lessening available iron. Interestingly, some chelators, e.g., deferiprone, may act by redistributing iron [123,124]. This effect could be of potential benefit, although ultimately having the redistribution result in improved availability of iron to support proper protein function, e.g., serving as a cofactor for enzymatic reactions, would be a key determinant for its usefulness. In summary, ensuring that protein activities, which rely on metals, are functioning normally within the CNS should improve neuronal health and help offset disease progression.

## Figures and Tables

**Table 1:** Initial Stage AD Olfactory Bulb vs. Control Olfactory Bulb *.

Total Number of Genes Analyzed: 30,259					
Number of Genes with ≥ 1.5 Fold Expression: 2860					
Number of Genes with ≤ 1.5 Fold Expression: 3307					
**BioProfiler Category**	**Number (#) of Genes in Category**	**# of Genes with ≥1.5× Expression (*p* ≤ 0.05)**	**Hypergeometric *p* Value**	**# of Genes with ≤1.5× Expression (*p* ≤ 0.05)**	**Hypergeometric *p* Value**
Alzheimer’s Disease	829	139	**1.58e-11**	121	**5.79e-4**
Anemia (primary inquiry)	909	179	**9.81e-22**	144	**3.02e-6**
Mitochondria	864	136	**1.96e-9**	128	**2.21e-4**
Hypoxia	122	10	7.27e-1	20	**4.21e-2**
Iron	194	25	6.92e-2	25	2.19e-1
Iron transport	24	3	3.99e-1	6	**4.05e-2**
Heme synthesis	31	3	5.72e-1	5	2.47e-1
					
**Iron responsive elements** [38]	**# of genes in category**	**# of genes with ≥1.5× expression (*p* ≤ 0.05)**	**Hypergeometric *p* value**	**# of genes with ≤1.5× expression (*p* ≤ 0.05)**	**Hypergeometric *p* value**
3′ IRE	1885	262	**7.04e-11**	382	**1.01e-34**
5′ IRE	697	106	**6.49e-7**	139	**1.30e-12**
HQ 3′ IRE	189	24	8.39e-2	43	**2.30e-6**
HQ 5′ IRE	66	11	**4.43e-2**	9	2.93e-1

**Human iron-proteome category**	**# of genes in category**	**# of genes with ≥1.5× expression (*p* ≤ 0.05)**	**Hypergeometric *p* value**	**# of genes with ≤1.5× expression (*p* ≤ 0.05)**	**Hypergeometric *p* value**
Proteins binding individual iron ions	137	18	9.54e-2	21	6.95e-2
Heme-binding proteins	173	25	**2.14e-2**	22	2.57e-1
Iron–sulfur proteins	68	12	**2.46e-2**	14	**1.41e-2**

**UniProt keyword**	**# of genes in category**	**# of genes with ≥1.5× expression (*p* ≤ 0.05)**	**Hypergeometric *p* value**	**# of genes with ≤1.5× expression (*p* ≤ 0.05)**	**Hypergeometric *p* value**
Iron KW-0408	344	48	**4.13e-3**	45	1.17e-1

* Analysis of dataset GSE93885 generated by Lachen-Montes et al. [33]. Bold *p* values indicate statistical significance.

**Table 2:** PITRM1 KO vs. WT Cerebral Organoids from Induced Pluripotent Stem Cells—All Clusters Combined *.

Total Number of Genes Analyzed: 19,561					
Number of Genes with ≥ 1.5 Fold Expression: 222					
Number of Genes with ≤ 1.5 Fold Expression: 228					
**BioProfiler Category**	**Number (#) of Genes in Category**	**# of Genes with ≥1.5× Expression (FDR ≤ 0.05)**	**Hypergeometric *p* Value**	**# of Genes with ≤1.5× Expression (FDR ≤ 0.05)**	**Hypergeometric *p* Value**
Alzheimer’s Disease	789	10	4.06e-1	23	**5.26e-5**
Anemia (primary inquiry)	879	16	**4.31e-2**	21	**1.51e-3**
Mitochondria	846	10	4.93e-1	18	**1.04e-2**
Hypoxia	117	0	1.00	2	3.97e-1
Iron	194	6	**2.36e-2**	4	1.91e-1
Iron transport	26	1	2.57e-1	0	1.00
Heme synthesis	32	0	1.00	1	3.13e-1
					
**Iron-responsive elements** [38]	**# of genes in category**	**# of genes with ≥1.5× expression (FDR ≤ 0.05)**	**Hypergeometric *p* value**	**# of genes with ≤1.5× expression (FDR ≤ 0.05)**	**Hypergeometric *p* value**
3′ IRE	1762	17	7.92e-1	23	3.15e-1
5′ IRE	638	14	**1.45e-2**	15	**8.07e-3**
HQ 3′ IRE	165	2	5.61e-1	5	**4.43e-2**
HQ 5′ IRE	57	2	1.37e-1	3	**2.88e-2**

**Human iron-proteome category**	**# of genes in category**	**# of genes with ≥1.5× expression (FDR ≤ 0.05)**	**Hypergeometric *p* value**	**# of genes with ≤1.5× expression (FDR ≤ 0.05)**	**Hypergeometric *p* value**
Proteins binding individual iron ions	135	2	4.54e-1	2	4.68e-1
Heme-binding proteins	163	1	8.46e-1	1	8.53e-1
Iron–sulfur proteins	65	0	1.00	0	1.00

**UniProt keyword**	**# of genes in category**	**# of genes with ≥1.5× expression (FDR ≤ 0.05)**	**Hypergeometric *p* value**	**# of genes with ≤1.5× expression (FDR ≤ 0.05)**	**Hypergeometric *p* value**
Iron KW-0408	317	1	9.74e-1	3	7.17e-1

* Derived from analysis of dataset GSE147047 generated by Pérez et al. [37]. Bold *p* values indicate statistical significance.

**Table 3:** PITRM1 KO vs. WT Cerebral Organoids from Induced Pluripotent Stem Cells—Individual Clusters *.

Cluster	Cell Type	Class of Genes Upregulated by ≥1.5× with Hypergeometric *p* ≤ 0.05	Class of Genes Downregulated by ≤1.5× with Hypergeometric *p* ≤ 0.05
0	Neurons	Anemia	Alzheimer’s; Anemia
1	Neurons	Anemia	-
2	Neurons	5′-IRE	-
3	Glia	-	Alzheimer’s; iron; 5′-IRE; Mitochondria; Proteins binding individual iron
4	Progenitor cells	-	-
5	Neurons	Anemia	-
6	Progenitor cells	-	Mitochondria
7	Neurons	Anemia	-
8	Neurons	Anemia	5′-IRE; HQ 5′-IRE
9	Astrocytes	-	-
10	Progenitor cells	Anemia	-
11	Glia	Anemia	-
12	Neurons	-	-
13	Astrocytes	HQ 3′-IRE	Alzheimer’s; Anemia; Mitochondria
14	Neurons	-	-
15	Neurons	-	-
16	Progenitor cells	Anemia	-
17	Neurons	-	5′-IRE; HQ 5′-IRE
18	Neurons	HQ 5′-IRE	3′-IRE; HQ 3′-IRE; Mitochondria; Heme synthesis
19	Neurons	-	-
20	Neurons	-	-
21	Neurons	-	-
22	Microglia	-	-
23	Glia	-	Alzheimer’s; Mitochondria
24	Progenitor cells	Anemia; Iron	5′-IRE
25	Microglia	-	-

* Derived from dataset GSE147047 generated by Pérez et al. [37].

**Table 4:** Overlapping expression of anemia-related genes between datasets *.

	BioProfiler Category	Genes with ≥1.5× in Common between Datasets	Hyper-Geometric *p* Value	Genes with ≤1.5× in Common between Datasets	Hyper-Geometric *p* Value	Genes with ≥1.5× or ≤1.5× in Common between Datasets	Hyper-Geometric *p* Value
**(Initial Stage AD Olfactory Bulb** vs. Control Olfactory Bulb) vs. (PITRM1 KO vs. WT Cerebral Organoids)	Anemia	ARG2, RPS29, SHMT2	3.16e-1	CTNNB1, FSTL1, HMGB1, MEIS2, SRGAP3	**4.96e-2**	ARG2, CTNNB1, FSTL1, GPI, HMGB1, MAPK10, MEIS2, RPS29, SHMT2, SRGAP3, THY1	1.02e-1
**(Middle Stage AD Olfactory Bulb** vs. Control Olfactory Bulb) vs. (PITRM1 KO vs. WT Cerebral Organoids)	Anemia	ARG2, SHMT2, THY1	2.13e-1	HMGB1, TUBA1A, TUBA1C	1.94 e-1	ARG2, GPI, HMGB1, KMT2C, MAPK10, PBX1, PGM3, RPS29, SHMT2, SRGAP3, THY1, TUBA1A, TUBA1C	**3.40e-3**
**(Advanced Stage AD Olfactory Bulb** vs. Control Olfactory Bulb) vs. (PITRM1 KO vs. WT Cerebral Organoids)	Anemia	SHMT2	8.69e-1	GPI, PBX1, SRGAP3	3.23e-1	CALR, FSTL1, GPI, PBX1, RPS10, RPS29, SHMT2, SRGAP3, TUBA1C	2.93e-1

* Dataset GSE93885 generated by Lachen-Montes et al. [33] and dataset GSE147047 generated by Pérez et al. [37]. Bold *p* values indicate statistical significance.

## Data Availability

Not applicable.

## Supplementary Materials

The following supporting information can be downloaded at: https://www.mdpi.com/article/10.3390/brainsci13030511/s1, Supplementary Materials.
